# Army News

**Published:** 1919-02

**Authors:** 


### ARMY NEWS

A place for the Hun!
    A place where no water or shade is;
A place in the sun
For the son-of-a-gun
    A million times hotter than Hades!

Dr. Alexis Carrel, wearing the uniform of major in the French army, arrived in New York on the steamship La Lorraine. Dr Carrel, who is connected with the Rockefeller Institute for Medical Research, has not yet been discharged from service.

Seventeen wounded and gassed soldiers from overseas arrived in San Antonio February 2nd from Hoboken, N. J., nine of them went to the Camp Travis Base Hospital and eight to the Fort Sam Houston Base Hospital. Those sent to Camp Travis are all from Texas.

Doctor George Carlisle, formerly of the Baylor Medical Unit which went to France in June, 1918, has returned to Dallas on his way to Fort Sam Houston. Dr. Carlisle was commissioned in the medical unit, but was transferred to another unit last October. Lieutenant Carlisle left France several weeks ago and landed in New York January 18.

Two hundred American Red Cross nurses died here and abroad of influenza contracted while nursing soldiers.

Doctor E. H. Cary, dean of the Baylor Medical college has received a letter from Major M. E. Lott, commanding officer of the Baylor Hospital Unit, stating that the unit had been ordered home. The letter was dated January 8, and Dr. Cary thinks the unit will reach America within the next few days. The unit is composed of about ninety persons, most of whom are from Dallas and North Texas.

A letter from Dr. Olin D. Wannamaker, of Dallas, Texas, to his wife, the first he has been able to write since he was seriously injured in an automobile accident near Padua, Italy, states that his condition was worse than the first diagnosis of his injuries showed. Several of the vertebrae of his spine have been badly injured. Italian doctors treating him say that it will take another month for him to recover.

Why Germany bombed hospitals is no longer a mystery. Gilbert Mayer of the 104th Field Signal Battalion, says he asked a German officer why his nation was violating the rules of civilized warfare in that respect, and Mayer asserts the officer replied: "It takes only a few months to make a fighting man and only a little longer to make an officer, but it takes seven years to make a doctor." So it was to kill doctors that the Huns kept this up, even into November, according to some soldiers here who were back in hospitals until the armistice was signed.

An official report from General Pershing made public January 26 by the War Department shows that on January 9 there were 105,753 men of the American Army in hospitals in France and England, of whom 72,642 were suffering from disease and 33,111 from wounds or other injuries.

